# The Influence of Spatial Configuration of Residential Area and Vector Populations on Dengue Incidence Patterns in an Individual-Level Transmission Model

**DOI:** 10.3390/ijerph14070792

**Published:** 2017-07-15

**Authors:** Jeon-Young Kang, Jared Aldstadt

**Affiliations:** Department of Geography, University at Buffalo, Buffalo, NY 14261, USA; geojared@buffalo.edu

**Keywords:** dengue, agent-based model, spatial configuration, mosquito population, serotype dominance

## Abstract

Dengue is a mosquito-borne infectious disease that is endemic in tropical and subtropical countries. Many individual-level simulation models have been developed to test hypotheses about dengue virus transmission. Often these efforts assume that human host and mosquito vector populations are randomly or uniformly distributed in the environment. Although, the movement of mosquitoes is affected by spatial configuration of buildings and mosquito populations are highly clustered in key buildings, little research has focused on the influence of the local built environment in dengue transmission models. We developed an agent-based model of dengue transmission in a village setting to test the importance of using realistic environments in individual-level models of dengue transmission. The results from one-way ANOVA analysis of simulations indicated that the differences between scenarios in terms of infection rates as well as serotype-specific dominance are statistically significant. Specifically, the infection rates in scenarios of a realistic environment are more variable than those of a synthetic spatial configuration. With respect to dengue serotype-specific cases, we found that a single dengue serotype is more often dominant in realistic environments than in synthetic environments. An agent-based approach allows a fine-scaled analysis of simulated dengue incidence patterns. The results provide a better understanding of the influence of spatial heterogeneity on dengue transmission at a local scale.

## 1. Introduction

Dengue is a significant and growing public health concern in tropical and subtropical developing countries. The World Health Organization (WHO) estimates that 50–100 million dengue infections, transmitted primarily by the *Aedes aegypti* mosquito, occur annually in the Asia-Pacific region [1]. It has particularly affected children under 15 years old in Thailand [2]. A dengue vaccine has been licensed for use in several countries, but in most affected countries the current efforts for dengue prevention and control focus on reducing mosquito population [3]. An improved understanding of the characteristics of dengue transmission can enhance the effectiveness of the prevention and control. In addition, it is difficult to precisely predict where and when dengue occurs because of its different propensity and severity of four distinct serotypes (DENV-1, DENV-2, DENV-3, and DENV-4), as well as seasonal fluctuation of dengue incidence [4,5,6].

As is well known, environmental factors influence the dynamic nature of dengue. In other words, the factors such as climate and land use/land cover impact the local ecology of *Ae. aegypti* [7,8,9,10,11,12,13], which in turn, influences the large scale spatio-temporal patterns of dengue occurrence. Additionally, *Ae. aegypti*’s movement is an important determinant of the local dynamics of dengue. These mosquitoes generally move between neighboring houses [14,15,16]. The vectors can spread DENV among nearby locations while they move around.

Recent studies often fail to describe dengue epidemics at a local scale since the factors that are associated with dengue are usually collected based on the aggregated units (e.g., census tracks, districts) [17,18]. This aggregation makes it impossible to study the within unit variation of infection risk and understand factors related to risk at the local level. Given spatially and temporally clustered patterns of dengue incidence [19,20,21] and local predominance of a specific serotype of dengue by year [22], aggregated data limits study of dengue virus transmission.

As an alternative approach, an agent-based model (ABM) is conducive to fully describe dynamic phenomena at the micro-scale by defining each heterogeneous agent, its behaviors, and interactions between agents and environments. Also, ABMs enable consideration of the impact of asymptomatic individuals, which may be impossible to be considered in the research based on data regarding hospitalized patients. ABMs have proved a useful way to integrate current knowledge of dengue transmission and address research questions via simulation [23,24,25,26,27]. They empirically support the claim that human movements have a significant influence on transmission of the virus [28]. So far, however, there has been little discussion in what ways a spatial configuration impacts dengue transmission patterns. In spite of the possibilities that the spatial distribution of a residential area can influence mosquito movement [14,29], there is a noticeable lack of research on this issue.

Interested in addressing this issue, the main objectives of this study are twofold: (1) to develop an agent-based model for dengue transmission and (2) to determine how the spatial configuration of residential buildings has an impact on dengue incidence patterns. Specifically, we seek to answer the following research questions:

- How does the spatial configuration of buildings influence dengue incidence rates?
- How does the spatial configuration impact serotype-specific dominance?
- How does mosquito population distribution influence dengue infection rates, as well as serotype-specific dominance?

These questions are answered through development and implementation of an ABM for dengue transmission. In this model, four environments were set up, where human and mosquito agents interact with each other in specific locations (e.g., houses, workplaces, and schools). As mosquito population structure is an important determinant for the epidemic evolution [30,31], we considered both heterogeneous and homogenous mosquito population. Ultimately, this paper compared four scenarios: (1) HeteroReal (Heterogeneous mosquito population in a realistic spatial configuration); (2) HomoReal (homogeneous mosquito population in a realistic spatial configuration); (3) HeteroSynth (heterogeneous mosquito population in a synthetic spatial configuration); and (4) HomoSynth (homogeneous mosquito population in a synthetic spatial configuration). In addition, as a measurement of spatial configuration effects and dengue serotype-specific dominance patterns, we used the Gini and Herfindahl indices, which have been widely used to describe the concentration of species at a specific space and time location. Through a one-way ANOVA analysis of such indices, this study illustrated the differences of dengue infection rates and dengue serotype-specific dominance patterns caused by spatial configurations of residential area.

## 2. Materials and Methods

### 2.1. Study Area and Data

**Figure 1:**
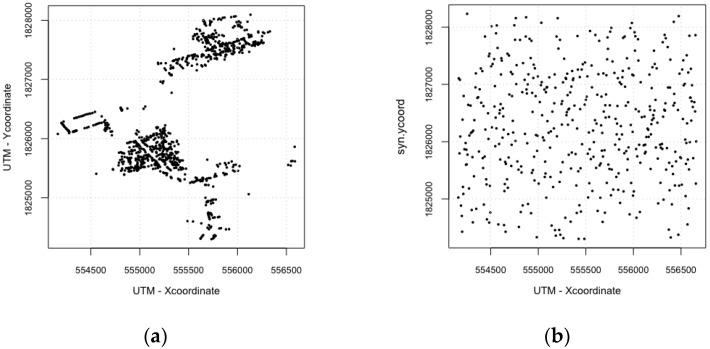
Spatial configurations in study area, a village, Kamphaeng Phet Province (KPP), Thailand (projected in Universal Transverse Mercator (UTM) coordinates). (**a**) Realistic spatial configuration; (**b**) Synthetic spatial configuration.

Our study area is based on a portion of Kamphaeng Phet Province (KPP), Thailand. In order to test spatial configuration effects on dengue occurrence patterns, realistic and synthetic environments were used. In the realistic model, all of the houses were located at a realistic location identified from GPS data, whereas in the synthetic configuration, they were randomly arranged (Figure 1). In Figure 1, the black dots represent houses, workplaces and schools.

**Figure 2:**
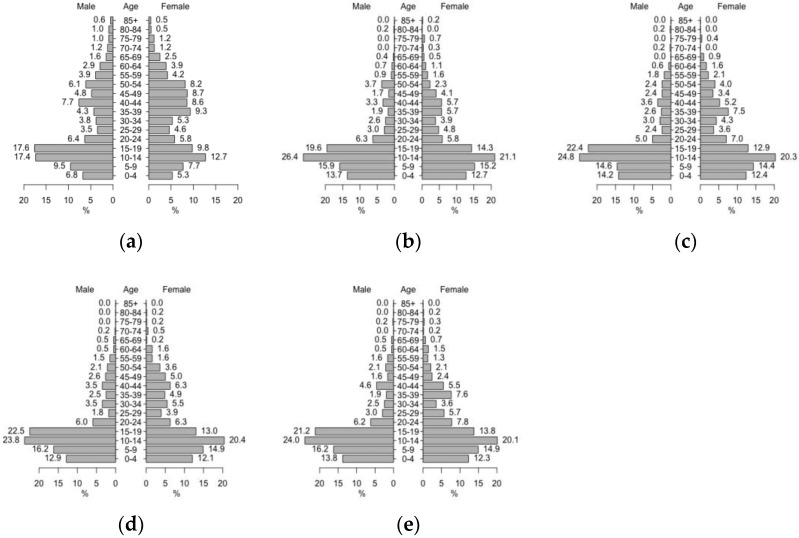
Synthesized population pyramid. (**a**) Total population; (**b**) Susceptible population to DENV-1; (**c**) Susceptible population to Denv-2; (**d**) Susceptible population to Denv-3; (**e**) Susceptible population to Denv-4.

In a realistic configuration, the houses, schools and workplaces were spatially closely located from northwest to southeast and northeast to southwest. In a synthetic configuration, all of buildings are evenly distributed. The environment of the model was composed of 895 houses, 20 workplaces, and 4 schools. We utilized parts of a registered residents dataset of KPP in 2009 [32], and the samples of households were drawn at random. The synthetic population had approximately 2800 individuals in 895 houses. The composition of synthesized population is shown in Figure 2a. In the study area, children aged 19 and under comprised approximately 50% of the total population. Figure 2b–e illustrate the susceptible population pyramids to specific serotypes (DENV-1, -2, -3, and -4), respectively. These greater numbers of children in a total susceptible population accurately represented that adults may have experienced greater numbers of dengue infections [19].

These experiments were undertaken within a period of one year, from 1 January 2014 to 1 January 2015. For a better estimation of the outputs, the simulations were run with 1000 iterations of each scenario. With the iterations, the simulations were executed over and over by using different experiment settings. In detail, compositions of household members were differently set up in every iteration for all scenarios, while locations of houses and mosquito populations varied based on the scenarios. Locations of houses were fixed in all iterations for scenarios of a realistic configuration while they were randomly arranged in iterations for synthetic configuration scenarios. For scenarios of homogeneous mosquito population, the mosquito population was fixed with 42 over iterations although it varied to each building over iterations for heterogeneous mosquito population scenarios.

### 2.2. Conceptual Model

**Figure 3:**
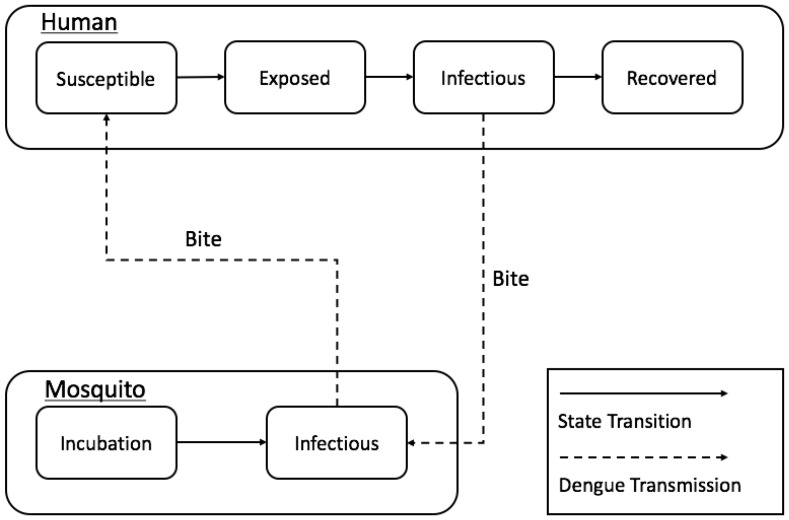
Flow diagram representing dengue transmission phases.

We adopted the Susceptible-Exposed-Infectious-Recovered (SEIR) model (Figure 3). When it comes to dengue infection process, typically people are in one of four states: ‘Susceptible’ (able to contract the virus), ‘Exposed’ (not yet infectious), ‘Infectious’ (able to transmit a disease) and ‘Recovered’ (immune to the virus). Dengue virus is interactively transmitted as the adult *Ae. aegypti* bites infected humans or the infected adult *Ae. aegypti* bites susceptible humans.

Due to serotype-specific immunity of dengue virus, each human agent has one state for each serotype. In other words, although a human may be immune to a specific serotype, they may still be susceptible to other serotypes. In addition, it is shown that cross-protection between serotypes is short-term [33,34,35,36]. In detail, a human infected with a certain serotype is not susceptible to other serotypes for two to three months.

### 2.3. Agent-Based Model

This paper developed an agent-based model of dengue virus transmission using Anylogic 7.3.5, a commonly used simulation platform. The model had three elements: (1) a set of agents, their attributes and behaviors; (2) a set of relationships between agents; and (3) the environment where agents interact with others. In detail, the agent refers to each human, infected female mosquito (the female mosquito becomes an agent when it gets infected), and building (house, school, and workplaces). The parameters in this model are largely the same as those of Chao, Halstead, Halloran and Longini Jr [23]. For the details, Table A1 provides the Overview, Design concepts, and Details (ODD) protocol originally proposed by Grimm, et al. [37].

**Figure 4:**
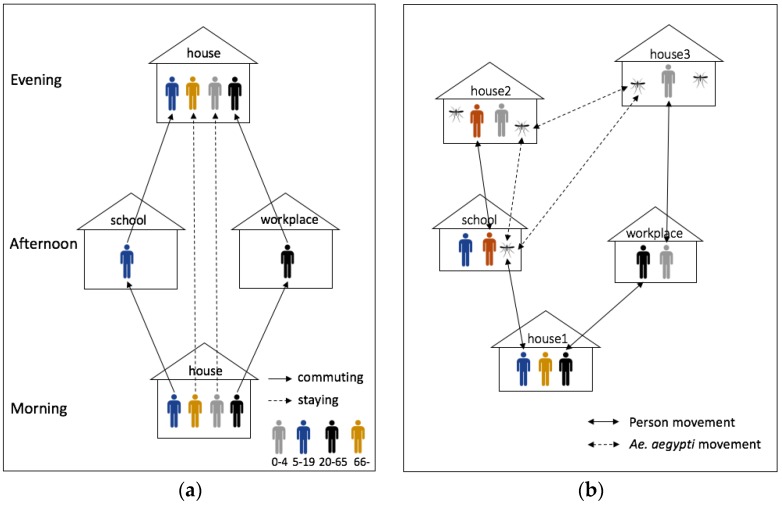
Human and *Ae. aegypti* movements. (**a**) Human movements in a spatio-temporal dimension; (**b**) *Ae. aegypti* movements in a spatial dimension.

When it comes to human agents, agent behaviors were defined as follows. The daily movement of humans depends on the age of each individual. An individual human spends the daytime (between 9 a.m. and 5 p.m.) at his/her workplace (ages 20 to 64) or school (ages 5 to 19), the morning (before 9 a.m.) and the nighttime (after 5 p.m.) at his/her home. Thus, people who commute to workplace/school interact with others in the workplace/school. The rest of the humans stay at their home all the time. The sick individual spends the whole day at his/her house until he/she recovers (Figure 4). Individual humans can be susceptible, exposed, infectious, or recovered to each dengue serotype. Heterotypic cross immunity lasts for 120 days. After 120 days, humans are again susceptible to serotypes that they have not yet been exposed to Vaughn, et al. [38]. Table 1 provides the parameters of an individual human agent, which are almost the same as Chao, Halstead, Halloran and Longini Jr [23]. All of parameters are the same in every scenario: HeteroReal, HomoReal, HeteroSynth, and HomoSynth.

**Figure 5:**
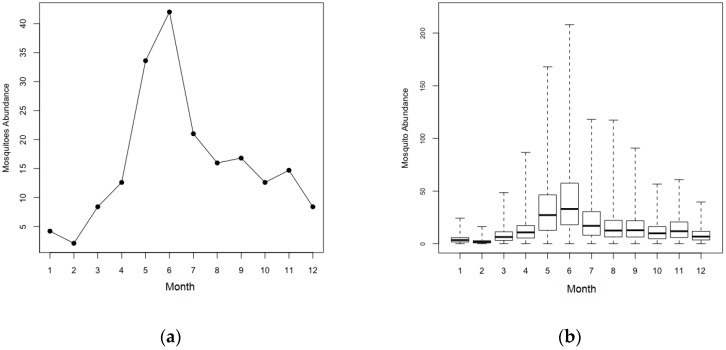
Mosquito seasonality. (**a**) The building-level mosquito abundance in homogenous mosquito population scenarios; (**b**) The mean and range of building-level mosquito abundance in heterogeneous mosquito population scenarios.

Homogeneous and heterogeneous mosquito population scenarios are distinguished in terms of the distribution of mosquito populations in the buildings, as shown in Figure 5. Since the number of mosquitoes vary seasonally with a peak in the hot and rainy summer season in Thailand [9], we assumed seasonal fluctuation of mosquito populations for both scenarios as in Chao, Halstead, Halloran and Longini Jr [23]. In the homogenous scenarios, the number of mosquitoes in each building is set to 42 at the peak in June, and the number of mosquitoes per buildings is just two in February (Figure 5a). *Ae*. *aegypti* populations are often highly aggregated or clustered at the building level [39,40]. In heterogeneous mosquito population scenarios, there is spatial variation in mosquito density in addition to seasonal fluctuation. Figure 5b shows the mean and the range of building-level mosquito populations for a single simulation in heterogeneous mosquito population scenarios. The bold lines denote the monthly average of building-level mosquito populations with the vertical dotted lines representing the range of values. The average number of mosquitoes in each building is approximately equal in all scenarios.

A mosquito can become infected when it bites an infectious human host. Only infected mosquitoes can transmit dengue virus to other susceptible humans. Infected and infectious mosquitos have a 0.15 probability of traveling to nearby buildings (<30 m) each day, as depicted in Figure 4b. To account for occasional long distance mosquito movements, there is also a 0.01 probability that mosquito agents travel to a randomly selected building that is farther than 30 m of its current buildings. We defined two hazard rates for mosquitoes depending on age, since the survival of mosquitoes is age-dependent [14,15,41]. We also defined mosquito’s biting rates at different times of day according to Chao, Halstead, Halloran and Longini Jr [23]. Table 2 shows the set of parameters of the mosquito agents. All of these parameters are identical in the all scenarios.

## 3. Results

### 3.1. Exploration on Infectious Rate

**Figure 6:**
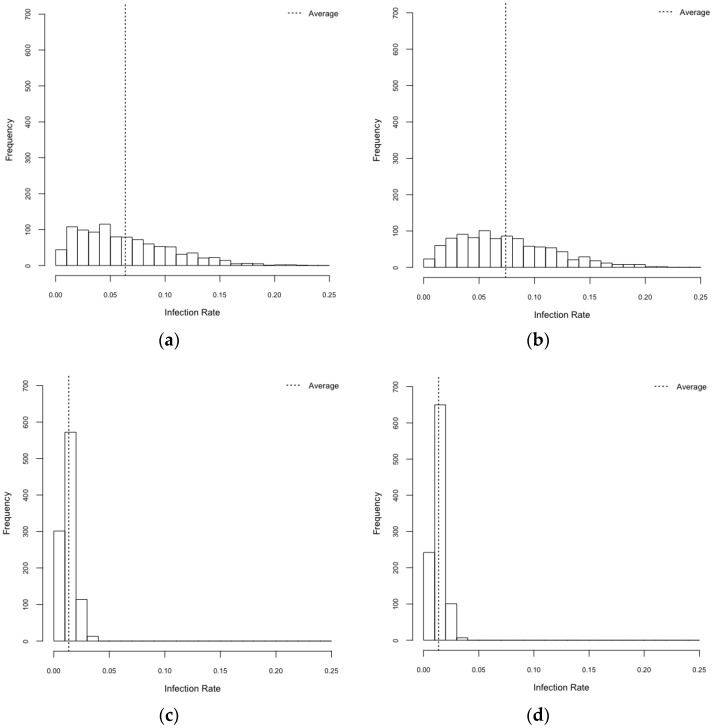
Infection rates. (**a**) HeteroReal; (**b**) HomoReal; (**c**) HeteroSynth; (**d**) HomoSynth.

We carried out four experimental scenarios: HeteroReal, HomoReal, HeteroSynth, and HomoSynth. Dengue virus infection rates varied considerably by scenarios (Figure 6 and Table 3). A one-way ANOVA indicated that there were significant differences in infection rates among HeteroReal (M = 0.064, SD = 0.042), HomoReal (M = 0.074, SD = 0.043), HeteroSynth (M = 0.013, SD = 0.006), and HomoSynth (M = 0.014, SD = 0.005), F_3,3996_ = 1125.7, *p* < 0.001. The infection rates were calculated as the number of total infection cases, including asymptomatic infections, per the number of total population. The result from the Bonferroni adjustment showed that infection rates in realistic environments were greater than in those in synthetic environments (*p* < 0.001).

Such greater infection rates in realistic environments were caused by the number of connected buildings. In our model, based upon mosquitoes’ movement radius, we defined connected buildings as those that are within the 30-m limit of regular mosquito movement. The more connected the buildings, the higher chance that infected mosquito vectors move between them. In other words, mosquitoes’ movements in realistic environments were less limited than those in synthetic environments (Table 4). This fits with the observation that dengue outbreaks are often spatially and temporally clustered [19,20,21].

**Figure 7:**
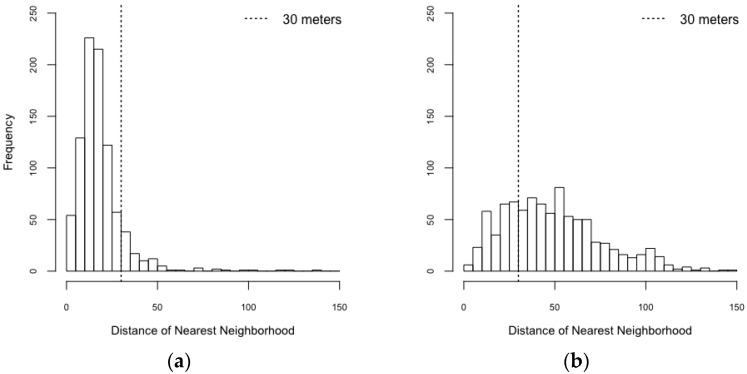
Distance of nearest neighborhood. (**a**) Realistic spatial configuration; (**b**) Synthetic spatial configuration.

Figure 7 shows distances of nearest neighbor to each house in the realistic and synthetic scenarios. Intuitively, the buildings in a realistic spatial configuration were more spatially clustered than those in a synthetic spatial configuration. The maximum distance of nearest neighborhood in the realistic and synthetic spatial configurations are 559.987 m and 167.423 m, respectively.

With respect to mosquito population influence, although homogeneous mosquito populations lead to more infections than heterogeneous mosquito population in realistic spatial configuration (*p* < 0.001), there was no difference of infection rates in synthetic spatial configuration (*p* > 0.05). In realistic configuration, the influence of the structure of mosquito population was statistically significant, and such influence was observed in previous studies [30,31]. There was not significant influence of mosquito population structure in synthetic environments and this may be attributed to insufficient dengue incidences (see Figure 6 and Table 3).

**Figure 8:**
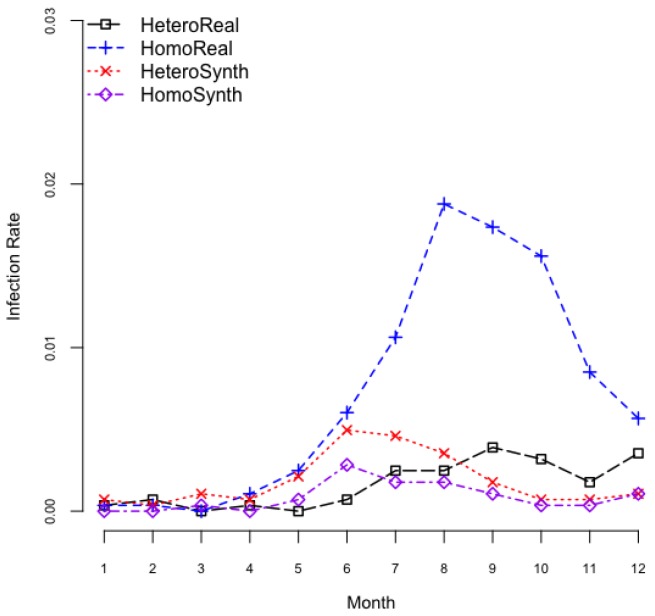
Infection rate variability.

Regardless of serotypes, overall monthly dengue virus infection rates of each scenario fluctuated from 0 to 0.04 (Figure 8). The infection rates were also seasonal, which can be accounted for by simply attributing the mosquito’s seasonal abundance [9]. Dengue infection rates were relatively higher in summer, and they are lower in winter. Interestingly, infection rates in homogeneous mosquito population in realistic environments were much greater than those in others.

### 3.2. Exploration on Dengue Serotype Dominance

When it comes to a specific-dengue serotype dominance, we employed two indices, the Gini index and the Herfindahl index. Both of these indices are used to measure concentration of values. Gini coefficient ranges from zero to one, with zero meaning that dengue infections are evenly distributed among the four serotype and one indicating that all dengue infections are due to the same serotype. The Herfindahl index is also ranged from zero to one, with zero meaning no concentration (even distributions of serotype-specific outbreaks) and one representing high concentration (serotype-specific dominance).

**Figure 9:**
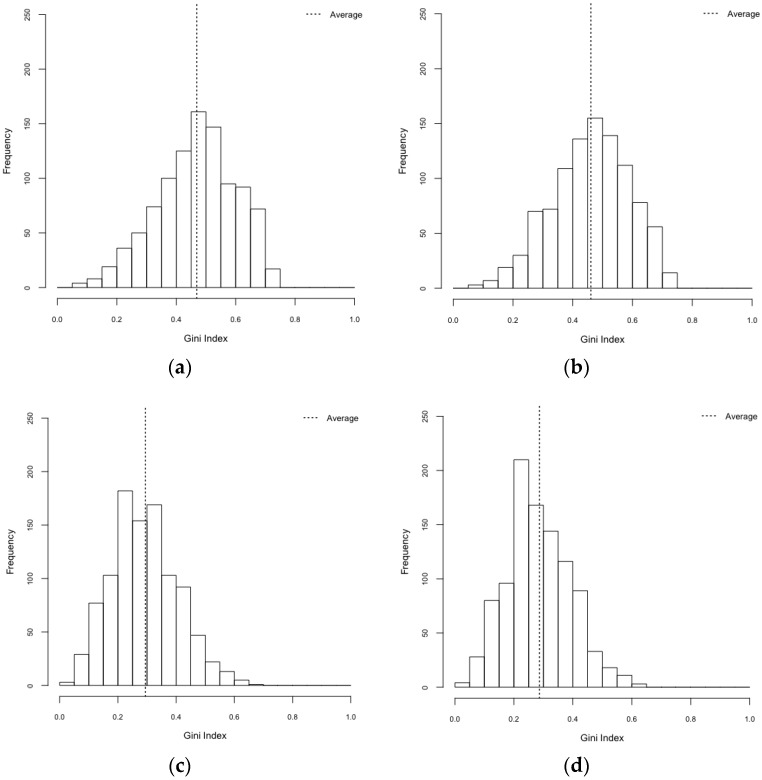
Gini index. (**a**) HeteroReal; (**b**) HomoReal; (**c**) HeteroSynth; (**d**) HomoSynth.

**Figure 10:**
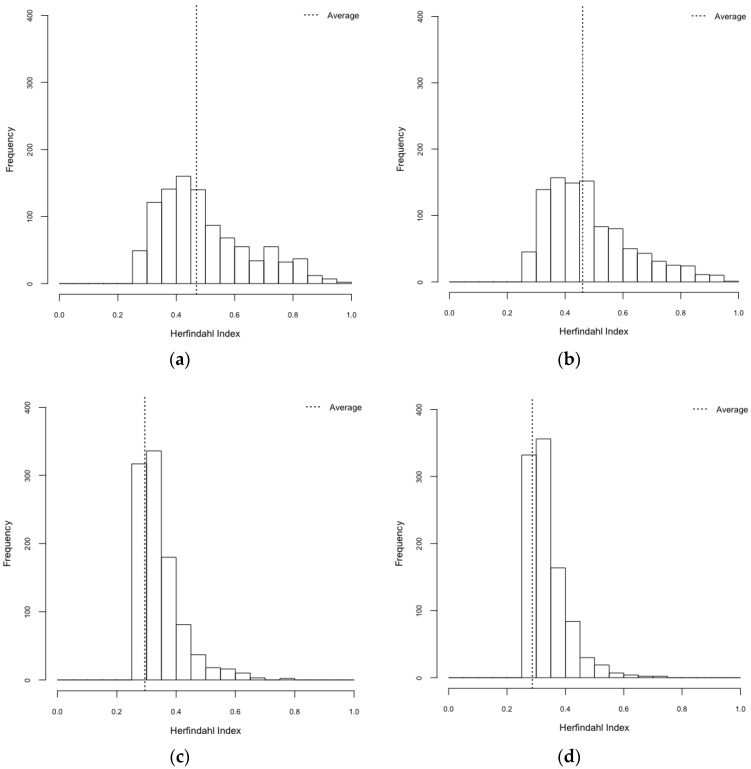
Herfindahl index. (**a**) HeteroReal; (**b**) HomoReal; (**c**) HeteroSynth; (**d**) HomoSynth.

A specific-serotype was more dominant in realistic environments than that in synthetic environments (Table 5, Figure 9 and Figure 10), which was statistically significant (F_3,3996_ = 689.2, *p* < 0.001 and F_3,3996_ = 533.08, *p* < 0.001 for Gini and Herfindahl indices, respectively). Post hoc analysis using Bonferroni illustrated that both Gini and Herfindahl indices in a realistic environment were greater than in those in a synthetic environment (*p* < 0.001). Put differently, we uncovered that the influences of spatial configuration and mosquito population heterogeneity on serotype-specific dominance are statistically significant. These findings yield a better understanding of dynamic nature of dengue outbreaks.

## 4. Conclusions

We examined the spatial configuration effects on dengue incidence patterns using an agent-based model. These experiments were motivated by the fact that mosquito movement is influenced by spatial configurations of buildings and mosquitoes’ habitats [14,29]. To provide a better understanding of such effects on dengue virus transmission, we developed a model in which individual humans and mosquitoes are thought of as agents. They move actively through the environment and interact with each other. To see the differences of outcomes by changes of spatial configuration of residential area and mosquitoes, we explored four scenarios: (1) HeteroReal; (2) HomoReal; (3) HeteroSynth; and (4) HomoSynth. One thousand iterations of each scenario were performed.

Through the results of ANOVA, we found the statistically significant differences between scenarios of the realistic environment and those of the synthetic environment in terms of dengue infection rates. The realistic environment had significantly higher infection rates than the synthetic environment. This may be attributed to the spatially clustered populations in the realistic environment. By employing Gini and Herfindahl indices, we also uncovered the differences of serotype-specific dominance in realistic environments between scenarios with heterogeneous mosquito populations and those of homogeneous mosquito distributions. Such concentration indices showed that individual serotypes were more likely to be dominant in the realistic environment compared to the synthetic environment.

This study sheds light on the importance of specifying the spatial configurations of the residential area. The results of our experiments showed a considerable influence of human residential and mosquito population patterns. Overall infection rates were significantly different between realistic and synthetic residential patterns. Local serotype dominance, which is commonly observed in dengue outbreaks, was more common when mosquito populations were heterogeneously distributed. These results reflect the importance of local ecology in dengue transmission and the recent shift in thinking from regional to local strategies for dengue surveillance and control [3]. Agent-based models are increasingly used to study disease control efforts in general, and the impacts of dengue vaccine distribution in particular. If these models are used to guide vaccine rollout then spatial configuration of human and vector populations should be seriously considered and included in the model sensitivity analysis. Agent-based models are often employed when local heterogeneities are thought to be important parts of a process, but improper specification of the local environment may limit their correctness and usefulness.

Although the study attempted to apply an agent-based model for dengue virus transmission, there is still room for improving the model. We did not take into consideration the demographic changes over time. To alleviate this problem, we explored a simulation within one year. As a consequence, this study was unable to examine annual variations in dengue infection patterns. In addition, this study area was relatively small, and thus it was not possible to examine spatial configuration effects on dengue infection patterns in large heterogeneous regions.

## Figures and Tables

**Table 1:** Set of parameters for human agents used to do experiments.

Parameters	Value	Note
Incubation period	6 days	Time between exposure and infectiousness
Viremic period	4 days	Time between infectious and recovered stages
Recovered period	120 days	Days of complete cross-immunity after recovery
P_MP_	0.25	Probability of mosquito to person transmission
P_PM_	0.1	Probability of person to mosquito transmission
Introduction rate	0.00001	Influx DENV from outside of study area
Infected rate	0.14	Annual infection rate used to simulate population immunity

**Table 2:** Set of parameters for mosquito agents used to do experiments.

Parameters	Value	Note
Movement probability	0.15, 0.01	Daily movement probability within neighbors and random locations
Movement radius	<30 m	Movement radius
Extrinsic incubation period	11 days	Days to become infectious
Hazard rate	0.09, 0.08	Younger than 10 days and older than 10 days
Biting rate	0.08, 0.76, 0.13, 0.03	Varies by time period (08–13, 13–18, 18–24, 00–08)

**Table 3:** Infection rates for each scenario.

Scenarios	Infection Rates (95% CI)
HeteroReal	0.064 (0.061–0.066)
HomoReal	0.074 (0.071–0.077)
HeteroSynth	0.013 (0.013–0.014)
HomoSynth	0.014 (0.013–0.014)

**Table 4:** Set of parameters for mosquito agents used to do experiments.

Spatial Configuration	Counts of Isolated Buildings	Counts of Connected Buildings
Realistic configuration	111	804
Synthetic configuration	693	222

**Table 5:** Gini and Herfindahl indices.

Scenarios	Gini Index (95% CI)	Herfindahl Index (95% CI)
HeteroReal	0.469 (0.461–0.477)	0.497 (0.487–0.506)
HomoReal	0.460 (0.452–0.469)	0.483 (0.473–0.492)
HeteroSynth	0.294 (0.287–0.301)	0.344 (0.339–0.348)
HomoSynth	0.287 (0.280–0.293)	0.337 (0.333–0.341)

## Abbreviations

Following abbreviations are used in this manuscript:

ABM:	Agent-Based Model
HeteroReal:	Heterogeneous mosquito population in a realistic spatial configuration
HomoReal:	Homogeneous mosquito population in a realistic spatial configuration
HeteroSynth:	Heterogeneous mosquito population in a synthetic spatial configuration
HomoSynth:	Homogeneous mosquito population in a synthetic spatial configuration
ODD:	Overview, Design concepts, and Details

## Appendix A

Table A1 describes Overview, Design concepts, and Details (ODD) protocol of ABM in this paper. Not applicable elements in ODD protocol introduced by Grimm, Berger, DeAngelis, Polhill, Giske and Railsback [37] are omitted.

**Table A1:** Overview, Design concepts and Details of ABM.

Overview	
Purpose	To simulate a local-level dengue transmission with four scenarios: (1) HeteroReal, (2) HomoReal, (3) HeteroSynth, and (4) HomoSynth
Entities, state variables, and scales	ABM consist of three entities: (1) human, (2) mosquito, and (3) building agents, and each entity has several state variables.
	(1) Human agent
	● Age
	● Gender
	● Occupation status
	● House location: x-y coordinates
	● School/workplace location: x-y coordinates
	● Current location: x-y coordinates
	● SEIR states for all DENV serotypes
	● Cross immunity state
	(2) Mosquito agent
	● Age
	● Serotype
	(3) Building agent
	● Type
	● Location: x-y coordinates
Process overview and scheduling	(1) Movement
	● Human: commuting process: school (aged 5–19) and workplace (aged 20–64)
	● Mosquito: moving around within 30 m (15% of probability) and random locations (1% of probability)
	(2) Biting
	● Mosquitoes bite humans with a certain probability.
	(3) Seasonal fluctuation of mosquito population
	● The counts of mosquito population vary by month.
**Design Concepts**	
Basic principles	Our model purposes to test hypothesis: (1) in what ways spatial configurations of buildings influence dengue transmission at a local scale; and (2) how the structure of a mosquito population affects dengue transmission at a local scale.
	The model was implemented based on Chao, Halstead, Halloran and Longini Jr [23]. A considerable difference between our model and Chao, Halstead, Halloran and Longini Jr [23] is the environment. Chao, Halstead, Halloran and Longini Jr [23] built the model based on grid spatial structures at which each grid (30 m^2^) has only one type of building, but our model represented each building as a point. Therefore, our model can have a finer scale to measure Euclidean distances for mosquito’s movements.
Sensing	Each mosquito senses the neighboring houses to move around and human to bite in all buildings.
Interaction	There is an interaction between humans and mosquitoes by biting process of mosquitoes.
**Details**	
Initialization	The model synthesizes human population within 895 households.
	Individual humans’ immune statuses to each serotype are assigned based on their ages with a certain probability (0.14).
	For scenarios of heterogeneous mosquito population, the populations are determined by a negative binomial distribution (0.0344, 1.5) where 0.0344 and 1.5 denote the number of failures and the probability of success.
	For scenarios of synthetic environments, all buildings are randomly arranged.
Input data	(1) locations of houses and schools identified from GPS data [32]
	(2) household census data [32]
Parameters	The parameters of human and mosquito agents were provided in Table 1 and Table 2.
